# Fluorinated Polydopamine Shell Decorated Fillers in Polytetrafluoroethylene Composite for Achieving Highly Reduced Coefficient of Thermal Expansion

**DOI:** 10.3390/polym16070987

**Published:** 2024-04-04

**Authors:** Yuanying Yu, Xiao Chen, Dajun Hou, Jingjing Zhou, Pengchao Zhang, Jie Shen, Jing Zhou

**Affiliations:** 1 State Key Laboratory of Advanced Technology for Materials Synthesis and Processing, School of Materials Science and Engineering, Wuhan University of Technology, Wuhan 430070, China; yyying0035@163.com (Y.Y.); xchen@whut.edu.cn (X.C.); hdj0068@163.com (D.H.); zhoujingjing@whut.edu.cn (J.Z.); shenjie@whut.edu.cn (J.S.); 2 Sanya Science and Education Innovation Park, Wuhan University of Technology, Sanya 572024, China; 3 Hubei Longzhong Laboratory, Wuhan University of Technology Xiangyang Demonstration Zone, Xiangyang 441000, China

**Keywords:** PTFE composites, polydopamine shell, thermal expansion, fluorine-containing groups, mechanical properties

## Abstract

The noticeable difference in the coefficient of thermal expansion (CTE) for polytetrafluoroethylene (PTFE) coatings and copper substrates is a major challenge for thermal debonding of the copper-clad laminate (CCL) in high-frequency communications. Theoretically, ceramic fillers with low CTEs in the coating can effectively reduce the gap, and there remains a trade-off between the dispersibility of fillers and the interfacial interactions with the polymeric matrix. Here, we propose a novel approach to prepare a pentafluorobenzoyl chloride (PFBC)-modified polydopamine (PDA) shell on silica particles by using amidation. Such modified particles perform excellent dispersion and exhibit diminished interfacial gaps in the PTFE matrix, which highly reduces CTE to 77 ppm/°C, accounting for only 48.1% of the neat coating. Moreover, the composite exhibits enhanced mechanical strength and toughness, and consequently suppresses thermal debonding in CCL under high-temperature conditions. Therefore, results present a promising potential for its use in the next-generation CCL of high-frequency communication devices.

## 1. Introduction

Copper-clad laminate (CCL) consists of a polytetrafluoroethylene (PTFE) coating and copper (Cu) foil substrate, which plays a vital role for manufacturing printed circuit boards (PCBs) for electronic devices [1]. However, they suffer severe damage resulting from the accumulated Joule heat, especially in the field of high-frequency communications with the applied electric energy increased to tens of GHz [2]. Specifically, one of the most serious hazards is the thermal debonding of the PTFE coating from the Cu substrate. Such phenomena are due to the large discrepancy between the coefficients of thermal expansion (CTEs) for PTFE (~109 ppm/°C) and Cu (~19 ppm/°C) [3]. Thus, the thermal debonding is usually caused by the mismatched thermal stresses generated from them when the PCB works long term under high-temperature conditions, thus diminishing their CTEs mismatch becomes essential to improve their performance.

The incorporation of ceramic fillers with low CTEs is the most frequently used strategy to achieve the goal of CTE reduction in PTFE coating [4,5]. However, the severe aggregation of fillers and poor interfacial interactions between fillers and PTFE are the two main challenges for the PTFE composites due to the ultra-low surface energy of PTFE [6]. It has been reported that various fillers decorated by hydrophilic surfactants have been employed to improve their dispersibility in PTFE aqueous emulsions for preparing the composite coating [7]. Although high loadings of the hydrophilic fillers are required to reduce the CTE, aggregates are easily generated to influence their performance, which presents a trade-off effect. Concomitantly, the enormous interfaces induced by the fillers will form a large number of defects and voids that disrupt the continuity of the PTFE phase, which deteriorate the dielectric constant (D_k_) and loss (D_f_) of the PTFE composites [3]. Such deterioration will result in enormous Joule heat when the electronic devices work at a high frequency, which accelerates the thermal debonding in CCL [8]. For the PTFE-based CCLs for high-frequency and high-speed applications, the focus is usually on the performance of low D_k_, low D_f_, and low CTEs of the composites. These three parameters are the main criteria for evaluating the practical application ability of PTFE-based CCLs [3,4]. Hence, it has been reported that enhancing the interfacial interaction between fillers and PTFE via decorating fillers with fluorosurfactants is an efficient way to suppress the deterioration, owing to the compatibility with PTFE [9,10]. However, it is still a challenge for maintaining uniform dispersion with hydroxyl groups while containing fluorinated functional groups on the fillers simultaneously to have synergies in low CTEs.

In the frame of this study, the low CTE silica particles (SiO_2_) are applied in the PTFE coating for addressing the trade-off of the dispersibility and the compatibility. The polydopamine (PDA) has been used as a hydrophilic platform for decorating SiO_2_ fillers, owing to the virtue of its strong adhesive property to inorganic and organic interfaces and tunable versatile functional groups [11]. Subsequently, pentafluorobenzoyl chloride (PFBC) is introduced on a PDA shell for grafting pentafluorophenone ring via amidation. The modified PDA shell contains both hydroxyl groups (-OH) from PDA chains and the introduced fluorine-containing groups, which improved dispersibility and compatibility simultaneously. As a proof of concept, the resulting composite PTFE controls the debonding defects during the high-temperature operations. Thus, such novel strategy suggested a promising pathway for suppressing thermal debonding in the CCL, promoting the development of high-performance PTFE composites in electronic devices.

## 2. Materials and Methods

### 2.1. Materials

Silica particles with an average diameter of ~1.49 μm were provided by Aladdin Industrial Corporation, Shanghai, China. Tris(hydroxymethyl) aminomethane (Tris, 99.8%), dopamine hydrochloride (98%), 2,3,4,5,6-Pentafluorobenzoyl chloride (PFBC, 99%), benzoyl chloride (BC, 99%), triethylamine (99%), iron chloride hexahydrate (FeCl_3_∙6H_2_O, 98%), and dichloromethane (DCM, 99.5%) were purchased from Aladdin Industrial Corporation, Shanghai, China. Polytetrafluoroethylene (PTFE) aqueous emulsion (60 wt%, TE-3865C) was purchased from Dupont Industrial Corporation, Wilmington, DE, USA.

### 2.2. Preparation of PFBC-D-SiO_2_ Fillers and CCL

The SiO_2_ modified by PDA and PFBC (abbreviated as PFBC-D-SiO_2_) fillers were prepared as follows: 2 g SiO_2_ powders was added into 200 mL of 1.2 mg/mL Tris solution and stirred for 20 min. The Tris solution was obtained by dissolving 240 mg of Tris powder into 200 mL of deionized water. Subsequently, 0.1 g dopamine hydrochloride was added into the solution and stirred for 24 h, and the obtained PDA-modified SiO_2_ (abbreviated as D-SiO_2_) sediments were washed with deionized water and dried for 60 °C. The dried sediments were dispersed in 30 mL of DCM, and 6 mL of triethylamine was added to the DCM solution and stirred for 10 min. An 80 μL volume of PFBC was then added to the solution and stirred at room temperature for 48 h. The modified particles were washed with ethanol and deionized water, and the PFBC-D-SiO_2_ slurry was obtained and then dried under vacuum at 60 °C for 12 h. As a control group of PFBC-D-SiO_2_, benzoyl chloride (BC)-modified D-SiO_2_ (abbreviated as BC-D-SiO_2_) was prepared via the same procedure used for preparing PFBC-D-SiO_2_.

The CCL was fabricated as follows: fillers were dispersed into the PTFE aqueous emulsion for fabricating the PTFE composites with a filler loading of 30 vol%. Subsequently, an appropriate amount of ethanol was added to the dispersion to demulsify the PTFE emulsion to form a doughy-like composite. The doughy-like composite was compressed by the three-bowl calendar repeatedly to form a lamina. The lamina was then dried in a vacuum oven at 180 °C for 48 h. Finally, the upper and lower sides of the dried lamina were attached with copper foils by hot-pressing to obtain the copper-clad laminate. The hot-pressing temperature was 385 °C for 2 h under pressure of 10 MPa in the vacuum.

### 2.3. Characterization

The microstructures of the fillers and the composite coatings were observed by high-resolution scanning transmission electron microscopy (STEM, Talos F200S, Waltham, MA, USA), scanning electron microscope (SEM, JSM-5610LV, Akishima, Tokyo, Japan), and metallurgical microscope (MJ 31, Hatagaya, Tokyo, Japan). The chemical analysis of the fillers was performed by Fourier transform infrared spectrometer (FTIR, Nexus, Gaithersburg, Maryland, USA) at the mid-infrared wavelength range of 400–4000 cm^−1^. X-ray photoelectron spectroscopy (XPS, ESCALAB 250Xi, Waltham, MA, USA) patterns of the fillers were obtained with Al Kα radiation (hν 1253.6 eV). The apparent contact angles of the water on fillers were determined by the optical contact angle measurement and the contour analysis systems (Data physics, OCA-20, Filderstadt, Germany) at room temperature and 60% relative humidity, the volume of water droplet was 5 μL, and five parallel samples were tested. The mechanical properties of the composites were assessed by an electronic universal testing machine (Instron 5967, Canton, MA, USA) with a drawing speed of 10 mm/min. Five duplicate samples were tested for every composite. The thermodynamic properties were revealed by dynamic mechanical analysis (DMA, DMA8000, Waltham, MA, USA) from 30 °C to 200 °C at the rate of 5 °C/min in air-conditioned environment. The coefficients of thermal expansion (CTEs) of the composites in the z-axis direction were revealed by Thermomechanical Analysis NETZSCH (TMA202, Selb, Germany) according to IPC-TM-650 2.4.41. All the samples were prepared with dimensions of 10 × 10 mm with the loading of 2 N, and the heating temperature ranged from 0 °C to 100 °C at a rate of 5 °C/min in the air-conditioned environment. The dielectric properties of the composites were measured by a microwave network analyzer (HP8722ET, Agilent, Palo Alto, CA, USA, @1–40 GHz) with the microstrip line method according to ICP-TM-650 2.5.5. All the composites were tailored to the size of 30 × 50 mm.

## 3. Results and Discussion

### 3.1. Surface Chemical Structure Analysis of the Fillers

**Figure 1:**
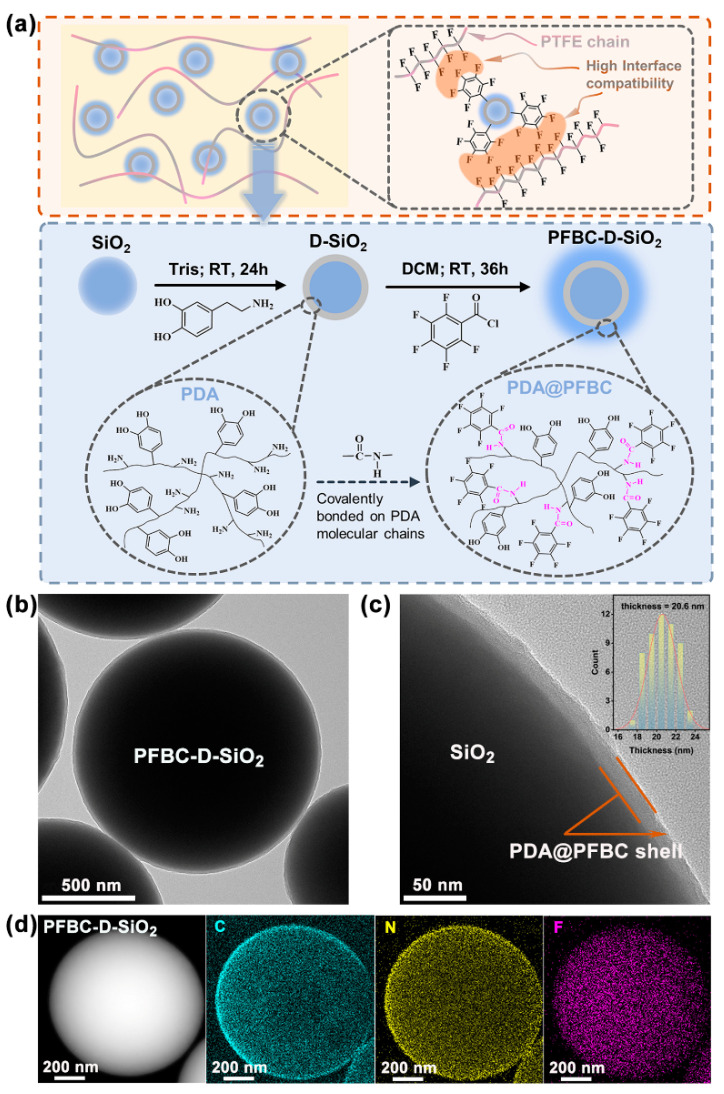
Microstructure and element component analyses of PFBC-D-SiO_2_: (**a**) schematic illustration of interfacial bonding between PFBC-D-SiO_2_ and PTFE chain and the synthesis of PFBC-D-SiO_2_; (**b**) the high-resolution TEM images of PFBC-D-SiO_2_; (**c**) the magnified TEM image of PFBC-D-SiO_2_, and the inset is the statistical analysis of the thickness of the PDA@PFBC polymer coating; and (**d**) high-angle annular dark field (HAADF) and EDS mapping of fluorine-containing-group-modified PDA shell.

To obtain PTFE composites with low CTEs, the fillers should have both low CTEs and D_k_ with D_f_, and SiO_2_ particles are excellent candidates with a CTE of 0.5 ppm/°C, D_k_ of 3.9, and D_f_ of 0.002 [3,10]. These favorable properties make SiO_2_ suitable for reducing the CTE of the PTFE composite. As it can be seen in Figure 1a, SiO_2_ particles are coated by PDA in advance via the polymerization of dopamine monomers at room temperature (RT) with the aid of Tris for 24 h. Subsequently, PFBC is adopted to react with the −NH_2_ of PDA to form the imide group (−CO−NH−) through amidation [12]. Hence, the pentafluorobenzene ring is covalently bonded with the PDA molecule chain. The fluorine-containing-group-modified PDA shell contains both hydrophilic groups with strong polarization (−OH, −CO−NH−, etc.) and hydrophobic groups with weak polarization (pentafluorobenzene ring). It can be found that a thin polymer shell (average thickness = 20.6 nm) is coated on the surface of the SiO_2_ particle in the local high-resolution TEM images of PFBC-D-SiO_2_ (Figure 1b,c). As shown in Figure 1d, C and N atoms are distributed on the surface of SiO_2_. Furthermore, a high amount of F atoms can also be detected on the surface of SiO_2_ originating from the introduced pentafluorobenzene ring. It demonstrates that the modified PDA shell (PDA@PFBC) exists well on the surface of SiO_2_ particles.

**Figure 2:**
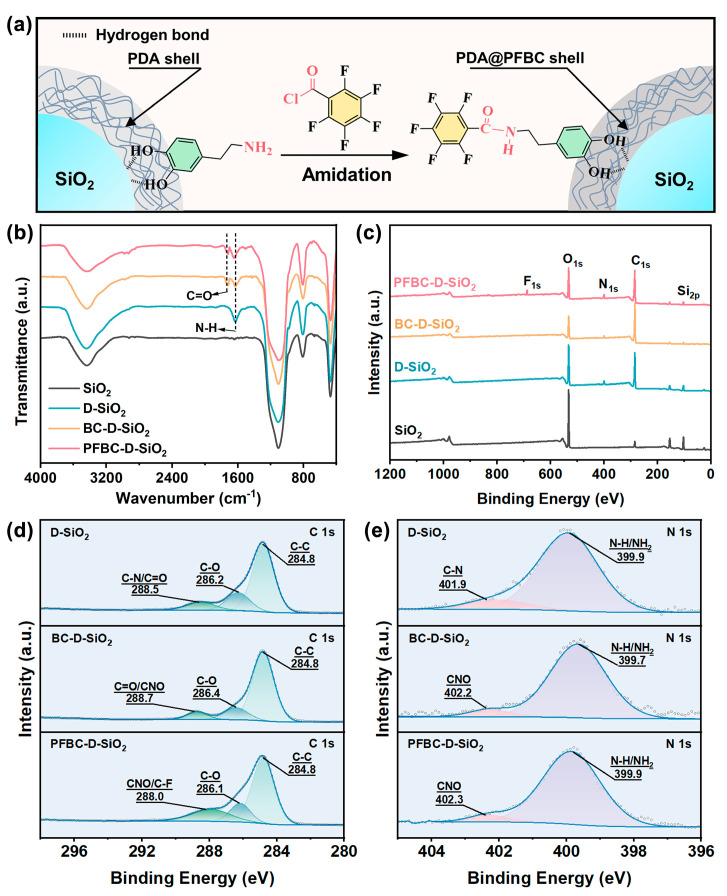
PDA shell modified by fluorine-containing group through the amidation reaction of −CO−Cl and −NH_2_: (**a**) the schematic illustration of amidation reaction between −CO−Cl of PFBC and −NH_2_ of PDA to form the modified PDA shell of PFBC-D-SiO_2_ and (**b**) FTIR and (**c**) XPS peak fitting of (**d**) C 1s and (**e**) N 1s HR−XPS spectra of SiO_2_, D-SiO_2_, BC-D-SiO_2_, and PFBC-D-SiO_2_.

Since the amidation reaction between −CO−Cl of PFBC and −NH_2_ of PDA is a unique characteristic occurring during the modification, −CO−NH− group can be utilized to verify the reaction (Figure 2a). The FTIR spectra of SiO_2_, D-SiO_2_, BC-D-SiO_2_, and PFBC-D-SiO_2_ are analyzed to investigate the generated −CO−NH− (Figure 2b). The peak at 3410 cm^−1^ corresponds to the stretching vibration of −OH originating from SiO_2_ [13]. The characteristic peaks of stretching vibration and flexural vibrations of O−Si−O bonds can also be detected separately at 1104 cm^−1^, 803 cm^−1^, and 475 cm^−1^ [14,15]. The observed peak at 1619 cm^−1^ in the curve of D-SiO_2_ originates from the shear vibration of amine group (−NH_2_) in PDA molecule chains [16]. All the characteristic peaks of BC-D-SiO_2_ and PFBC-D-SiO_2_ shifted to high-energy regions compared to those of D-SiO_2_. In addition, new peaks appear in the curves of BC-D-SiO_2_ and PFBC-D-SiO_2_, including 1710 cm^−1^ in both BC-D-SiO_2_ and PFBC-D-SiO_2_, originating from the stretching vibration of C=O group originating from the −CO−NH− [17].

Moreover, Figure 2c presents the chemical bonding and elemental species transformation due to amidation. The characteristic peaks at 101.5 eV and 532.5 eV in the four curves represented Si 2p and O 1s, respectively, which originate from SiO_2_ [18,19]. The peak intensity ratio of Si 2p and O 1s shows the obvious transformations after SiO_2_ is modified by PDA, PFBC, and BC, owing to the introduction of O atoms from these coating. Comparatively, a character peak of N (1s = 397.5 eV) originating from the PDA shell can be observed clearly in the curves of D-SiO_2_, BC-D-SiO_2_, and PFBC-D-SiO_2_ [20]. Nevertheless, a peak of F (1s = 687.9 eV) can also be detected in the curve of PFBC-D-SiO_2_ [21].

Furthermore, amidation is investigated by the peak fitting of C 1s (Figure 2d) and N 1s (Figure 2e) HR-XPS spectra of D-SiO_2_, BC-D-SiO_2_, and PFBC-D-SiO_2_. The curve fitting of the C 1s for D-SiO_2_ particles can be divided into three locations at 284.8 eV, 286.2 eV, and 288.5 eV, representing the C−C, C−O, and C−N or C=O bonding, respectively [20,22,23]. The C−C bonding corresponds to the benzene ring in the PDA molecule, and the C-N bonding is contributed by the amine group of PDA. The bonding of C−O and C=O is formed because the phenolic hydroxyl groups in the PDA molecule chains are oxidized and their hydrogen atoms are lost [24]. Comparingly, a new −CNO− bonding can be acquired by curve fitting the C 1s spectra of BC-D-SiO_2_ and PFBC-D-SiO_2_. Moreover, a new C−F bonding can only be obtained in the curve fitting of the C 1s for PFBC-D-SiO_2_. These transformations in the chemical environment of C atoms in PDA indicate that BC and PFBC can be covalently bonded with the PDA molecule chains.

In addition, the curve fitting for the N 1s spectra of D-SiO_2_, BC-D-SiO_2_, and PFBC-D-SiO_2_ is conducted (Figure 2e). The N 1s spectrum of D-SiO_2_ is curve-fitted into N−H or NH_2_ and C−N bonding at 399.9 eV and 401.9 eV, respectively, which originate from PDA [17,22]. After further modification, the −CNO− in the N 1s spectra of BC-D-SiO_2_ and PFBC-D-SiO_2_ appear at 402.2 eV and 402.3 eV, respectively [25]. Therefore, the curve fitting for the C 1s and N 1s spectra suggests that the chemical environment of the PDA shell is changed after it is modified by PFBC via amidation.

### 3.2. Wettability and Dispersibility of the Fillers

**Figure 3:**
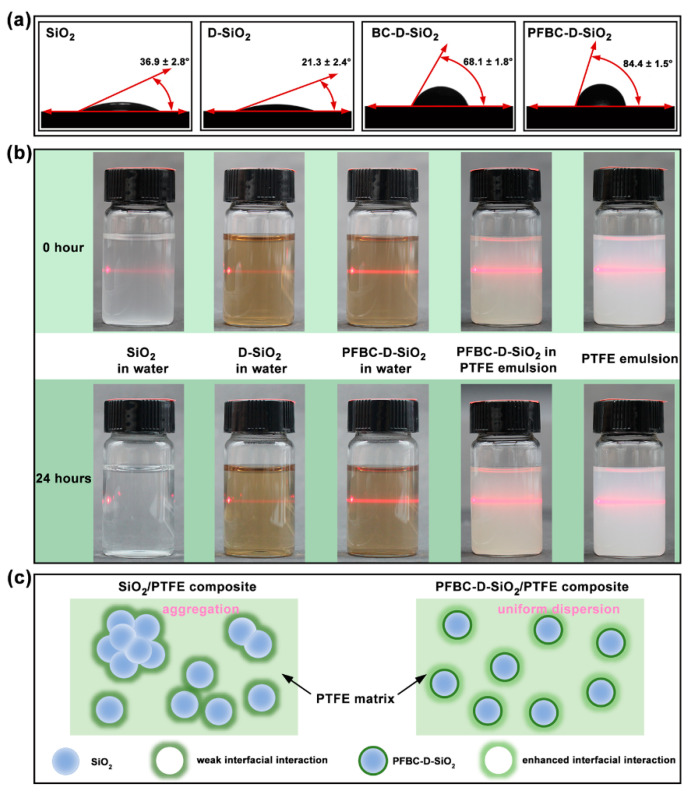
Dispersibility and static contact angles of the water on fillers: (**a**) images of the static contact angles of SiO_2_, D-SiO_2_, BC-D-SiO_2_, and PFBC-D-SiO_2_; (**b**) 24 h sedimentation experiment and the corresponding Tyndall effect test of the fillers in water and PTFE emulsion; and (**c**) illustration of the uniform dispersion of fillers and improved interfacial interaction between fillers and the PTFE matrix generated by the fluorine-containing-group-modified PDA shell.

To further access the chemical environment variation in the modified PDA shell, the wettability is performed in Figure 3a, which presents the static contact angles of the water on the fillers. In comparison with SiO_2_ (θ = 36.9 ± 2.8 °), the θ of D-SiO_2_ has reduced to 21.3 ± 2.4 ° owing to the highly hydrophilic PDA shell [26]. A 3~5 times increase in θ is obtained from BC-D-SiO_2_ and PFBC-D-SiO_2_ compared to D-SiO_2_, especially the θ of PFBC-D-SiO_2_ reaches 84.4 ± 1.5 °. It demonstrates that PFBC-D-SiO_2_ has successfully introduced the fluorinated functional groups on the particle surface against the hydrophilicity of the PDA. According to the composition analysis, the contact angle results agree with the coexistence of the pentafluorobenzene ring groups and hydroxyl groups in the PDA@PFBC shell.

To estimate the dispersible stability after filler incorporation, a 24 h sedimentation experiment of the fillers in water is conducted, which is combined with the observation of the Tyndall effect of these dispersion solutions (Figure 3b). At the beginning of the experiment (0 h), all dispersions show an obvious Tyndall effect that exhibits a bright red-light path through the solutions in vials. After standing for 24 h, SiO_2_ particles settled to the bottom of the vial and almost lost the Tyndall effect, while the red-light path in the D-SiO_2_ aqueous solution remains clear and bright. This phenomenon indicates that the D-SiO_2_ presents a strong hydrophilicity and results in a dispersion stability [27,28]. As for the PFBC-D-SiO_2_ aqueous solution, it displays a clear and bright red-light path after 24 h as well as 0 h status. It indicates that PFBC-D-SiO_2_ fillers have the ability to disperse stably in water [28,29]. Furthermore, the PFBC-D-SiO_2_ particles in the PTFE aqueous emulsion indicate the transformation of the red-light path during sedimentation. Similar to the phenomenon of PFBC-D-SiO_2_ in water, the red-light path remains bright after 24 h of sedimentation, demonstrating the excellent dispersibility in the PTFE aqueous emulsion. Therefore, Figure 3c illustrates that the PFBC-D-SiO_2_ fillers can be dispersed uniformly in the PTFE composites and can interact efficiently with the PTFE matrix.

### 3.3. Interfacial Bonding State of the PTFE Composites

**Figure 4:**
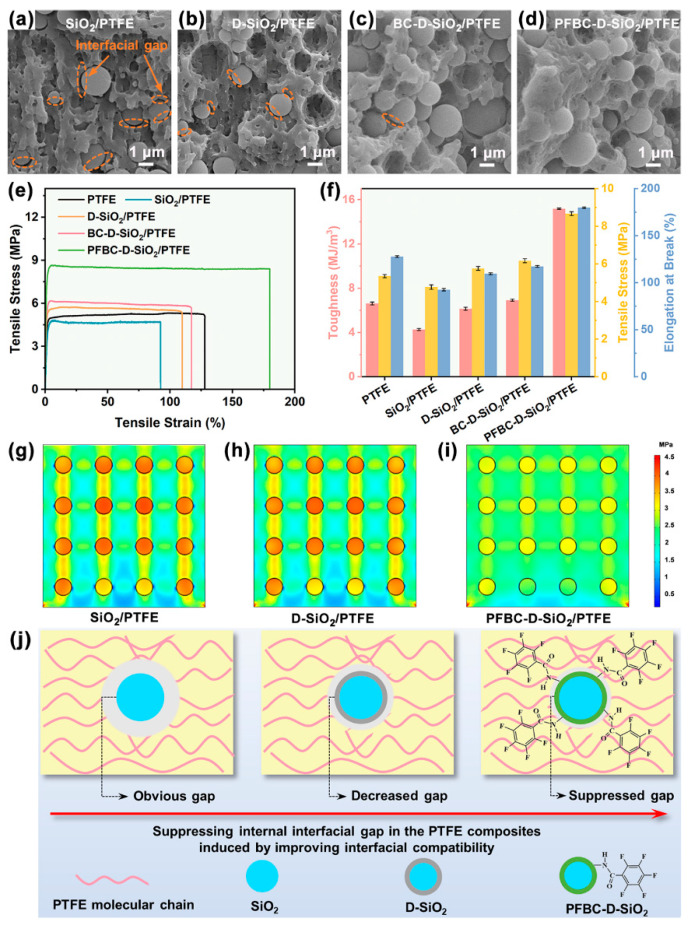
Interfacial bonding states of the interface between fillers and the PTFE matrix. SEM micrographs of the brittle-fractured cross-sections of the (**a**) SiO_2_/PTFE, (**b**) D-SiO_2_/PTFE, (**c**) BC-D-SiO_2_/PTFE, and (**d**) PFBC-D-SiO_2_/PTFE composites with 30 vol% of fillers; (**e**) stress–strain curves and (**f**) the corresponding toughness, tensile stress, and elongation at break of the PTFE composites with 30 vol% of fillers. The stress distribution model of (**g**) SiO_2_/PTFE, (**h**) D-SiO_2_/PTFE, and (**i**) PFBC-D-SiO_2_/PTFE under the vertically upward load calculated by COMSOL; and (**j**) illustration of the suppressed internal interfacial gap in the PTFE composites induced by improving the interfacial compatibility between fillers and the PTFE matrix.

It has been reported that the optimized loading content of D-SiO_2_ is 30 vol% for achieving the PTFE composite with the optimal CTE, which is also selected in this approach [18]. To observe the interfacial bonding states of the interfaces between fillers and the PTFE matrix, the PTFE composites were obtained by dissolving the copper foil of CCL in 0.5 g/mL of FeCl_3_ solution at 70 °C (Figure S1). The SEM micrographs of the cross-sections of the PTFE composites are shown in Figure 4a–d, in which the interfacial gaps are clear and large between SiO_2_ and the PTFE matrix as well as for D-SiO_2_. Although the highly hydrophilic D-SiO_2_ fillers can be dispersed well in the PTFE matrix, the apparent interfacial gaps remain in the composite. After being modified by the weakly polarized BC, the interfacial gaps between fillers and the PTFE matrix are suppressed in the BC-D-SiO_2_/PTFE composite (Figure 4c). Moreover, the interfacial gaps in PFBC-D-SiO_2_/PTFE composite are diminished due to the weakly polarized pentafluorobenzene ring group on the particles, resulting in its improved compatibility [30,31,32].

There is a direct relation between the interfacial bonding between fillers and polymer matrix and mechanical properties of the polymer-based composite [33]. Analyzing the stress–strain curves presented in Figure 4e, the PFBC-D-SiO_2_/PTFE composite demonstrates a tensile stress of 8.67 MPa, nearly 1.6 times higher than that of PTFE. Furthermore, the toughness of PFBC-D-SiO_2_/PTFE is 2.3 times greater than that of PTFE (Figure 4f). Compared to its counterparts, PFBC-D-SiO_2_/PTFE exhibits the highest mechanical properties. These improvements can be attributed to the enhanced interfacial bonding between PFBC-D-SiO_2_ and the PTFE matrix induced by the fluorinated PDA shell. Poor interfacial bonding disrupts the continuity of the PTFE phase, resulting in reduced toughness and elongation at break in PTFE composites containing SiO_2_, D-SiO_2_, and BC-D-SiO_2_ [34,35]. The significantly increased elongation at break of PFBC-D-SiO_2_ (180%), 1.4 times that of PTFE, can also be attributed to the enhanced interfacial bonding within the composite, allowing the fillers to effectively bridge the PTFE phase and further improve the composite’s stretch limit. Therefore, the strong interface zone acts as a stress buffer component inside the PFBC-D-SiO_2_/PTFE composite, enhancing its mechanical performance.

Finite element simulation was conducted by the software COMSOL 6.0 for visualizing the inner stress distribution inside the PTFE composites (Figure 4g–i). The parameters and boundary conditions are provided in the supporting information (Figure S2). It can be found that a large stress discrepancy between SiO_2_ fillers and the PTFE matrix is observed in Figure 4g, in which the stress concentration effect is released with low efficiency in the D-SiO_2_/PTFE composite (Figure 4h). Nevertheless, stress is distributed homogeneously inside the PFBC-D-SiO_2_/PTFE composite under the applied tensile stress (Figure 4i). With the interfacial gaps suppressed by the enhanced interfacial bonding between fillers and the polymer matrix (Figure 4j), the strong interfacial zones between PFBC-D-SiO_2_ fillers and the PTFE matrix can transmit the applied tensile stress smoothly to the SiO_2_ core [36]. In addition, the interfacial bonding status for the composite can also influence the total CTEs that related to the thermal debonding [4].

### 3.4. Mechanism of the Suppression of Thermal Debonding of PTFE Composites

**Figure 5:**
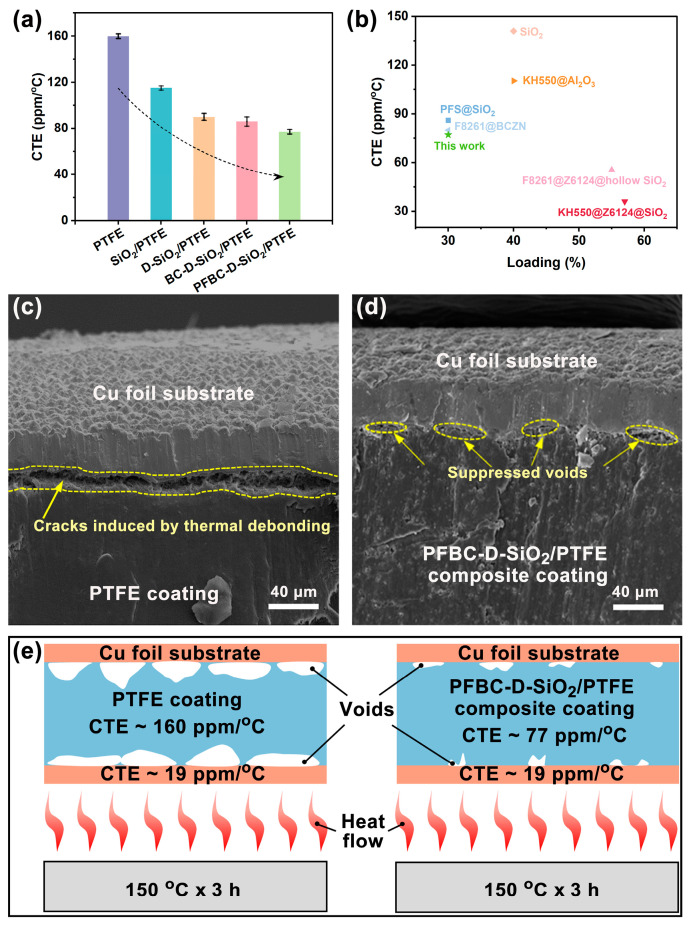
Suppressed thermal debonding in CCL induced by the PTFE composites: (**a**) CTEs of neat PTFE and the PTFE composites with 30 vol% of fillers; (**b**) comparison between this study and the reported studies in the aspects of CTE and filler loading; thermal debonding images in CCL containing (**c**) neat PTFE coating and (**d**) PFBC-D-SiO_2_/PTFE composite coating; and (**e**) mechanism of the thermal debonding in CCL under high-temperature operation conditions.

Therefore, it can be seen that Figure 5a presents a highly reduced CTE for the PFBC-D-SiO_2_/PTFE composite. The CTE of pure PTFE reaches up to 160 ppm/°C, owing to the severe volume expansion resulting from the molecule chain torsion [37]. In comparison with the neat PTFE, the CTEs of SiO_2_/PTFE, D-SiO_2_/PTFE, and BC-D-SiO_2_/PTFE are 115 ppm/°C, 90 ppm/°C, and 86 ppm/°C, respectively. Moreover, the CTE of PFBC-D-SiO_2_/PTFE decreases to 77 ppm/°C, which was 0.48 times that of the neat PTFE. The highly reduced CTE of PFBC-D-SiO_2_/PTFE can be attributed to the improved interfacial compatibility generated by the modified PDA@PFBC shell on the surface of SiO_2_ particles. This improved the interfacial compatibility, leading to the enhanced interfacial interaction between fillers and the PTFE matrix, which blocks the movement of PTFE molecules surrounding the PFBC-D-SiO_2_ fillers [4]. Such influence can also be supported by the highest storage modulus (E′) and lowest damping factor (Tan δ) of PFBC-D-SiO_2_/PTFE compared to those of the other counterparts (Figure S3) [38,39]. Furthermore, the strategy of the fluorinated PDA shell shows higher efficiency in reducing the CTE of the PTFE composite than that reported in previous studies (Figure 5b) [4,9,10,40,41,42].

To evaluate the long-term thermal stability of the PTFE-based CCLs, the thermal debonding test under high temperature over a long period and the dielectric test were conducted. The thermal debonding behavior of CCL was observed with the sandwiched CCLs (Figure S4) containing the neat PTFE coating and the PFBC-D-SiO_2_/PTFE composite coating with heating at 150 °C for 3 h. It can be found that cracks in the CCL containing the neat PTFE coating appear due to the thermal debonding of the coating from the Cu foil substrate (Figure 5c), whereas only a few microvoids can be detected (Figure 5d), indicating that the thermal debonding between the PFBC-D-SiO_2_ composite coating and the Cu foil substrate is efficiently suppressed. The difference in the thermal debonding state between the PTFE coating and the PFBC-D-SiO_2_/PTFE coating can be distinguished well after they are heated. The efficiently suppressed thermal debonding behavior can be attributed to the highly reduced discrepancy of CTE of both the composite coating and the Cu foil substrate, as illustrated in Figure 5e. On the other hand, dielectric performances should also be considered because the PTFE-based CCLs with D_k_ and D_f_ will generate Joule heat inside the high-frequency and high-speed communication electronic equipment. The D_k_ and D_f_ of the PFBC-D-SiO_2_/PTFE composite are reduced to 2.24 and 0.0015 at 30 GHz, respectively (Figures S5 and S6). The low D_k_ and D_f_ can inhibit the generation of Joule heat and synergistically suppress the thermal debonding in CCL as well. Therefore, the PFBC-D-SiO_2_/PTFE composite exhibits an efficient reduction in CTEs with an improved mechanical performance, indicating it to be an excellent candidate for CCL in high-frequency communications (Figure S7).

## 4. Conclusions

In this study, the fluorinated PDA shell was employed to modify the surface of SiO_2_ particles for the PFBC-D-SiO_2_ for achieving a uniform dispersion and a compatible interaction in the PTFE matrix. The uniform dispersion in the composite was achieved due to the hydroxyl groups provided by the PDA grafted on SiO_2_ particles. The improved interfacial compatibility between fillers and the PTFE matrix was generated by the additional fluorinated groups from the PFBC-modified PDA shell, which enhanced the interfacial interaction in the composite. The tensile stress and toughness of the PFBC-D-SiO_2_/PTFE composite were raised to 8.67 MPa and 15.19 MJ/m^3^, which are ~1.6 times and ~2.3 times higher than those of neat PTFE, respectively. Consequently, the resulting CTE of the composite was successfully decreased to 77 ppm/°C from 160 ppm/°C of neat PTFE. Owing to the decreased discrepancy of CTEs of the PTFE composite coating and the Cu foil substrate, the thermal debonding of the coating from the substrate in CCL was suppressed efficiently, while the D_k_ and D_f_ were low as well. Therefore, the fluorinated PDA shell on the fillers is a promising strategy to promote the development of PTFE composites in the field of high-frequency information transmission.

## Data Availability

Data are contained within the article.

## Supplementary Materials

The following supporting information can be downloaded at: https://www.mdpi.com/article/10.3390/polym16070987/s1, Figure S1: Photograph of etching process of CCL (a) and photographs of the composite before and after etching (b); Figure S2: Boundary conditions of the finite element simulation; Figure S3: Dynamic mechanical analysis for storage modulus curves (a) and Tan δ curves (b) of neat PTFE, SiO_2_/PTFE, D-SiO_2_/PTFE, BC-D-SiO_2_/PTFE, and PFBC-D-SiO_2_/PTFE exfoliated composite coatings with 30 vol% of filler loading content; Figure S4: (a) Cross-sectional images of CCL (inset is the optical image of the surface of CCL) and (b) schematic illustration of CCL shows its sandwich structure; Figure S5: Frequency dependence of dielectric constant (a) and loss (b) of neat PTFE, the exfoliated SiO_2_/PTFE, D-SiO_2_/PTFE, BC-D-SiO_2_/PTFE, and PFBC-D-SiO_2_/PTFE composite coatings with 30 vol% of filler loading content; Figure S6: Dielectric properties (@ 30 GHz) of neat PTFE, SiO_2_/PTFE, D-SiO_2_/PTFE, BC-D-SiO_2_/PTFE, and PFBC-D-SiO_2_/PTFE exfoliated composite coatings with 30 vol% of filler loading content; Figure S7: In comparison to the dielectric constant (D_k_), dielectric loss (D_f_), CTE, elongation at break, and toughness of neat PTFE, D-SiO_2_/PTFE, and PFBC-D-SiO_2_/PTFE exfoliated composite coatings with 30 vol% of filler loading contents. (Parameters of neat PTFE: D_k_ = 2.08, D_f_ = 0.00171, CTE = 160 ppm/°C, elongation at break = 92.36%, and toughness = 5.33 MJ/m^3^.)
