# Supplementary material for: Fluorinated Polydopamine Shell Decorated Fillers in Polytetrafluoroethylene Composite for Achieving Highly Reduced Coefficient of Thermal Expansion
Source: Polymers (Basel). 2024 Apr 4;16(7):987. doi: 10.3390/polym16070987 (PMC11014320; doi:10.3390/polym16070987)
Supplement: Supplementary file 1 [file polymers-16-00987-s001.zip › polymers-2947478-SI.pdf]

Supporting Information

Fluorinated Polydopamine Shell Decorated Fillers in Polytetrafluoroethylene Composite for Achieving Highly Reduced Coefficient of Thermal Expansion

Yuanying Yu [1,†], Xiao Chen [1,2,†], Dajun Hou [1], Jingjing Zhou [1], Pengchao Zhang [1,2,3,*], Jie Shen [1] and Jing Zhou [1,2,3,*]

[1] State Key Laboratory of Advanced Technology for Materials Synthesis and Processing, School of Materials Science and Engineering, Wuhan University of Technology, Wuhan 430070, China;
yyying0035@163.com (Y.Y.); xchen@whut.edu.cn (X.C.); hdj0068@163.com (D.H.); zhoujingjing@whut.edu.cn (J.Z.); shenjie@whut.edu.cn (J.S.)

[2] Sanya Science and Education Innovation Park, Wuhan University of Technology, Sanya 572024, China

[3] Hubei Longzhong Laboratory, Wuhan University of Technology Xiangyang Demonstration Zone,
Xiangyang 441000, China

* Correspondence: pczhang@whut.edu.cn (P.Z.); zhoujing@whut.edu.cn (J.Z.)

† These authors contributed equally to this work.

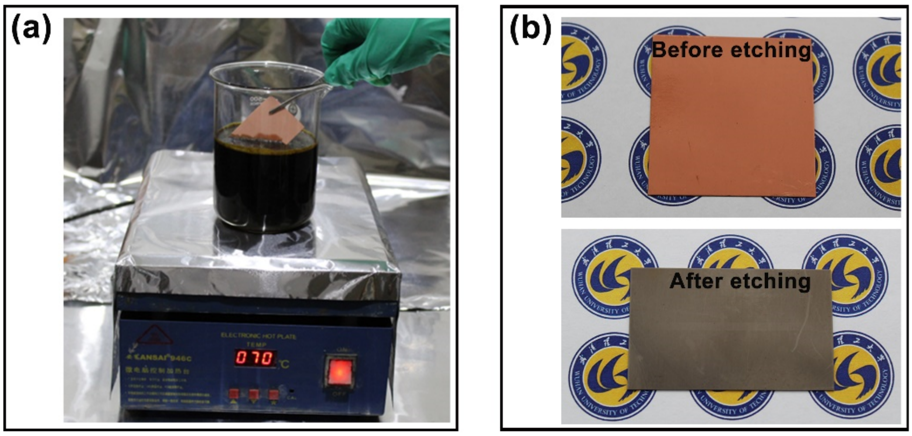

Figure S1. Photograph of etching process of CCL (a) and photographs of the composite before and after etching (b).

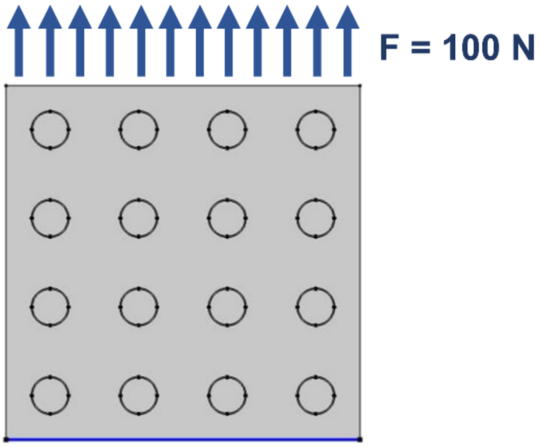

Figure S2. Boundary conditions of the finite element simulation.

In this study, the PTFE-based composite was established as a two-dimensional square model with a side length of 14 μm. According to the volume percentage of fillers, a 4 x 4 array of SiO_2 spheres with a radius (*r*). The *r* was established in the middle of the model, and the shell thickness (*d*) was established on the outside of the sphere. Correspondingly, the values of *r* and *d* were determined. The *r* is approximately 0.75 μm, and *d* is 0.02 μm determined by the TEM results (Figure 1). The PTFE-based composite was set as a linear elastic material. The boundary conditions were set beneath the model conditions. And a force of 100 N in the Y direction was applied to the upper section, as shown in Figure S2. By setting the boundary conditions, the stress distribution of the established two-dimensional model could be calculated under the applied force.

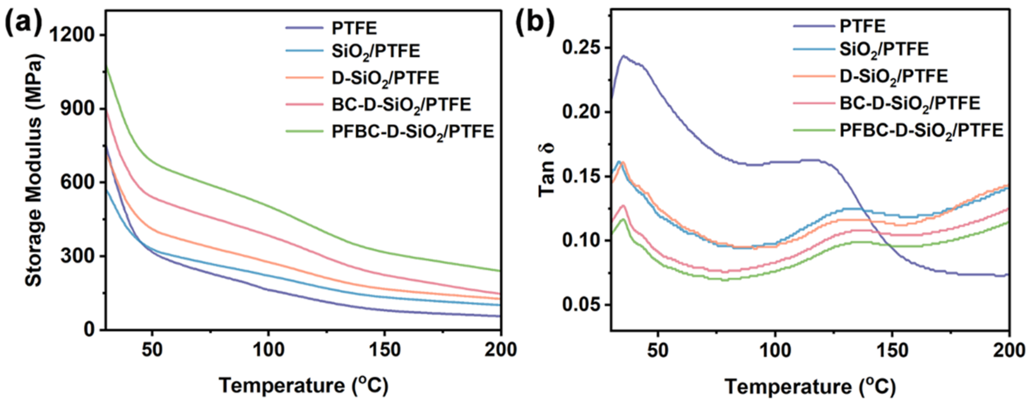

Figure S3. Dynamic mechanical analysis for storage modulus curves (a) and Tan δ curves (b) of neat PTFE, SiO_2/PTFE, D-SiO_2/PTFE, BC-D-SiO_2/PTFE, and PFBC-D-SiO_2/PTFE exfoliated composite coatings with 30 vol% of filler loading content.

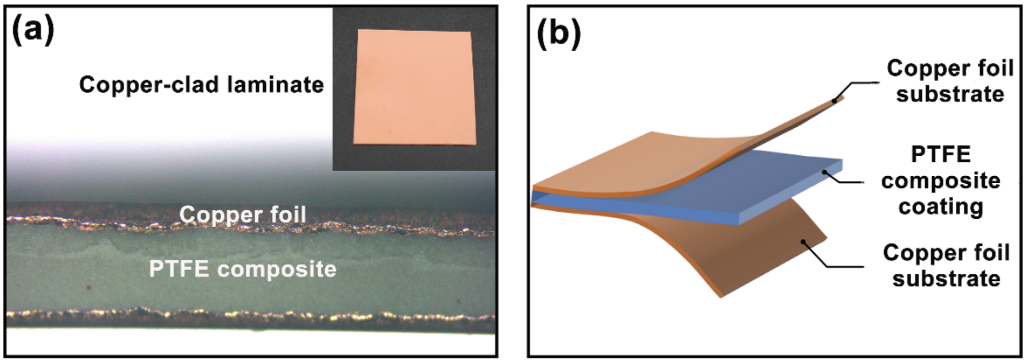

Figure S4. (a) Cross-sectional images of CCL (inset is the optical image of the surface of CCL) and (b) schematic illustration of CCL shows its sandwich structure.

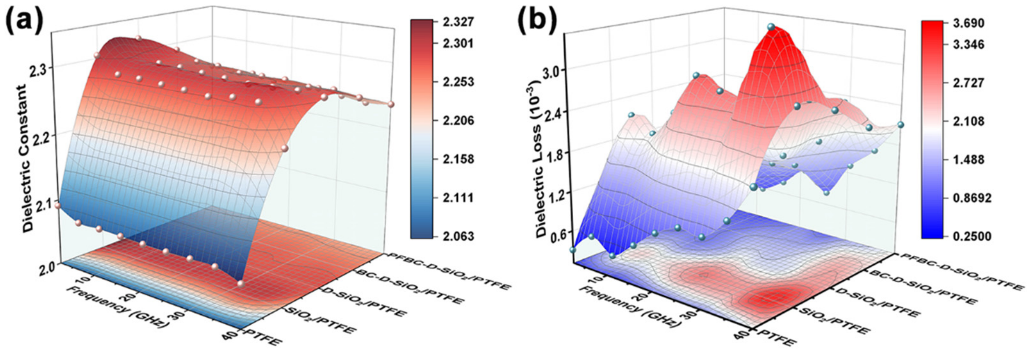

Figure S5. Frequency dependence of dielectric constant (a) and loss (b) of neat PTFE, the exfoliated SiO_2/PTFE, D-SiO_2/PTFE, BC-D-SiO_2/PTFE, and PFBC-D-SiO_2/PTFE composite coatings with 30 vol% of filler loading content.

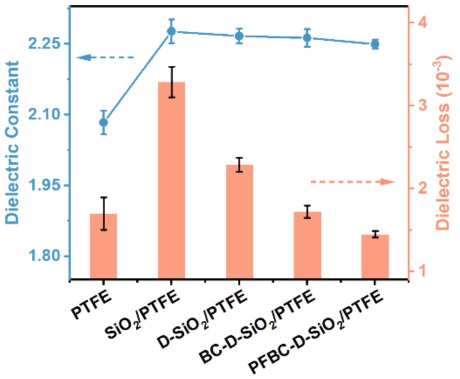

Figure S6. Dielectric properties (@ 30 GHz) of neat PTFE, SiO_2/PTFE, D-SiO_2/PTFE, BC-D-SiO_2/PTFE, and PFBC-D-SiO_2/PTFE exfoliated composite coatings with 30 vol% of filler loading content.

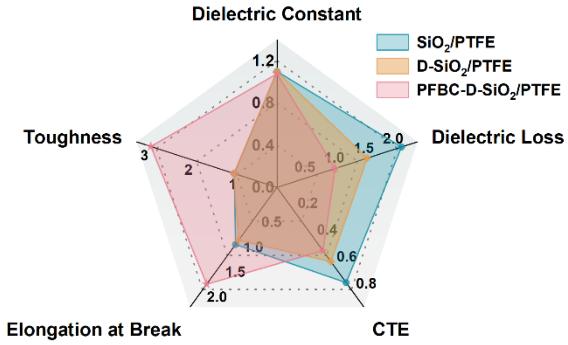

Figure S7. In comparison to the dielectric constant (D_k), dielectric loss (D_f), CTE, elongation at break, and toughness of neat PTFE, D-SiO_2/PTFE, and PFBC-D-SiO_2/PTFE exfoliated composite coatings with 30 vol% of filler loading contents. (Parameters of neat PTFE: D_k = 2.08, D_f = 0.00171, CTE = 160 ppm/°C, elongation at break = 92.36 %, and toughness = 5.33 MJ/m^3.)
